# The effect of online and in-person team-based learning (TBL) on undergraduate endocrinology teaching during COVID-19 pandemic

**DOI:** 10.1186/s12909-022-03173-5

**Published:** 2022-02-22

**Authors:** Shafeena Anas, Ioannis Kyrou, Mariann Rand-Weaver, Emmanouil Karteris

**Affiliations:** 1 College of Health, Medicine and Life Sciences, Brunel University London, Uxbridge, UB8 3PH UK; 2 Warwickshire Institute for the Study of Diabetes, Endocrinology and Metabolism (WISDEM), University Hospitals Coventry and Warwickshire NHS Trust, Coventry, CV2 2DX UK; 3 Centre for Sport, Exercise and Life Sciences, Research Institute for Health & Wellbeing, Coventry University, Coventry, CV1 5FB UK; 4 Aston Medical Research Institute, Aston Medical School, College of Health and Life Sciences, Aston University, Birmingham, B4 7ET UK; 5 Warwick Medical School, University of Warwick, Coventry, CV4 7AL UK; 6 Division of Thoracic Surgery, The Royal Brompton & Harefield NHS Foundation Trust, Harefield Hospital, London, UB9 6JH UK

**Keywords:** iRAT, tRAT, Application exercise, Endocrine disorders, Higher education, Mariann Rand-Weaver and Emmanouil Karteris should be considered joint last authors.

## Abstract

**Background:**

Team-based learning (TBL) combines active and collaborative learning, while incorporating aspects of the flipped classroom approach and problem-based learning. The COVID-19 pandemic presented certain challenges in the delivery of TBL in class. In this study, we investigated the impact of TBL on the academic performance of final year Biomedical Sciences’ undergraduate students in the context of an “Endocrine Disorders” study block. We did so by comparing the classical in-person approach and online delivery due to the COVID-19 pandemic.

**Methods:**

A non-compulsory TBL session was introduced to the curriculum of this block, which followed the traditional 2-h lecture delivery. Comparative analysis was performed for the exam and coursework performance of students who attended the TBL sessions (online and in-person) and those that did not.

**Results:**

Both cohorts of students who attended either in-person (*n* = 66) or online TBL sessions (*n* = 109) performed significantly better in their exams (*p* < 0.05) and a related coursework (*p* < 0.001 and p < 0.05, respectively) when compared to those that did not attend. For both these cohorts the exam mark distribution was much narrower compared to those that did not attend the TBL sessions where the majority of fails and “no shows” were recorded.

**Conclusions:**

Online and in-person TBL, can successfully supplement traditional lecture-based teaching and enhance the learning/performance, for complex medical subjects/topics. Our findings demonstrate that it is possible to deliver these sessions online with demonstrable benefit for students suggesting that there is greater flexibility in the use of TBL in higher education.

## Introduction

In biomedical/life sciences, senior undergraduate students are expected to gain an in depth understanding of clinically relevant complex subjects/topics, such as endocrine disorders (e.g. study the complex actions of hormones in health and disease). However, since there are no specific undergraduate degree courses in endocrinology, undergraduate biomedical students can undertake this subject through a study block as part of a variety of biosciences courses (e.g. biology, biochemistry, molecular biology, physiology, and neuroscience). Such clinically-oriented study blocks can be challenging, since students need to not only obtain comprehensive knowledge of the topic (e.g. knowledge of relevant terminology, clinical/analytical biochemistry, physiology and anatomy, and cell biology), but also develop robust understanding of the subject in its wider clinical context. In addition, cognitive skills, as well as other attributes, have to be further cultivated.

Emerging studies argue for adoption of different pedagogical approaches in Higher Education (HE), including flipped classroom learning, team-based learning (TBL), lab gamification, and online teaching material with asynchronous delivery [1, 2]. In biomedical sciences, students are further expected to think critically, and apply their knowledge in complex clinically relevant situations; hence structured support for active learning is needed [3]. Active learning focuses on the process of engaging students in activities that force them to reflect upon knowledge/ideas and how these should be applied [4], and may include a wide repertoire of approaches, ranging from group work with Q&A sessions to flipped classrooms, problem-based learning, and TBL [5]. Interestingly, a large-scale meta-analysis of data from 225 studies demonstrated that active learning in undergraduate science, technology, engineering and maths (STEM) programmes improves examination performance (by 6% on average), whilst is also linked to lower failure rates (average failure rates decrease from 34% with traditional lecturing to 22% with active learning, with students in traditional lecture-based courses being 1.5 times more likely to fail) [5, 6].

TBL is a pedagogical approach that combines both active and collaborative learning, and incorporates elements of the flipped classroom approach and problem-based learning (PBL) [7, 8]. Briefly, TBL is based on four underlying principles: (i) groups should be properly formed (teams are fixed for the whole course); (ii) students are accountable for their pre-learning and for working in teams; (iii) team assignments must promote both learning and team development; and (iv) students must receive frequent feedback [8]. In HE, TBL has shown to drive better engagement in the class, promote team participation, improve knowledge acquisition, and increase overall student satisfaction [9, 10].

In general, the benefits of applying TBL approaches in health science courses are well documented [9]. For example, multiple benefits have been reported from TBL for teaching applied pathophysiology to student nurses [11]. Indeed, a systematic study of the published literature on TBL in health professions education (HPE) reported that the vast majority of the papers studying the impact of TBL on learning outcomes concluded that TBL was an effective approach [10]. However, there is still limited research into the application of TBL for endocrinology, a complex and clinically relevant subject, in the curriculum of undergraduate HPE. To date, one US study looked at the use of TBL in an endocrine module as part of a pharmacy curriculum, reporting higher course grades for TBL module delivery compared to the traditional lecture-based approach [10]. Another study in China involving fourth-year medical students enrolled in an endocrinology internship showed that the performance of a group of students who followed a flipped classroom and PBL approach was better compared to the performance of those in a traditional lecture-based classroom, although the former also involved an increased workload for students [12]. It is evident that many dimensions/aspects of TBL have not been well-studied so far, particularly in the curriculum of undergraduate biomedical sciences (e.g. if enhanced engagement in class through TBL translates into better exam performance).

The Coronavirus Disease 2019 (COVID-19) has appeared in December 2019 and has been characterized as the first pandemic caused by a coronavirus [13, 14]. In order to contain the spread of COVID-19, widespread physical separation measures and movement limitations were implemented by many governments and authorities all over the word [15–17]. The COVID-19 outbreak is having a prolonged disrupting impact on people’s lives, including education, business, and the economy, as well as social life, politics, and entertainment [18]. Indeed, numerous digital technologies have been adopted in an attempt to resolve issues stemming from the pandemic. These range from telemedicine, to machine learning and cloud computing [19–22]. In higher education new approaches to learning and teaching had to be introduced, including the adaptation of TBL from in-person to online sessions [23, 24].

In the present study, we sought to investigate whether TBL can be incorporated (in-person or online) in a complex study block on endocrine disorders, if there are benefits from this approach for the learning experience/performance of undergraduate biomedical students, and whether the mode of delivery during the COVID-19 pandemic impacts on the overall performance.

## Methodology

### Study block

At Brunel University London, final year Biomedical Sciences’ undergraduate students have the opportunity to take an optional study block on “*Endocrine Disorders*” in order to study the pathophysiology and treatment of a selected range of clinically relevant, frequent endocrine diseases, using a case-based problem-solving approach. All methods were carried out in accordance with relevant guidelines and regulations.

### Case study cohorts

Final year undergraduate students registered on the “Endocrine Disorders” study block as part of the BSc Degree in Biomedical Sciences at Brunel University London. The two cohorts that were followed attempted TBL sessions in-person (2019–2020) and online (2020–2021). For the 2020–2021 cohort, due to the impact of the COVID-19 pandemic, the teaching of this block was redesigned towards a blended learning approach with a mixture of in-person and online teaching. As such, the applied TBL component of teaching was shifted entirely online for the 2020–2021 academic year. To facilitate this, a dedicated software for TBL was used, with all elements of the TBL phases including iRAT, tRAT and the application exercises delivered synchronously online. No need for specific ethical approval was required for the present analyses, since all relevant educational data were anonymised and available within Brunel University London. Data analyses presented here are part of teaching quality assurance exercise and anonymous student feedback rather an independent research study and performed in accordance with relevant guidelines and regulations. Subsequently, no further ethical approval is needed from our institution.

### Procedure

**Fig. 1:**
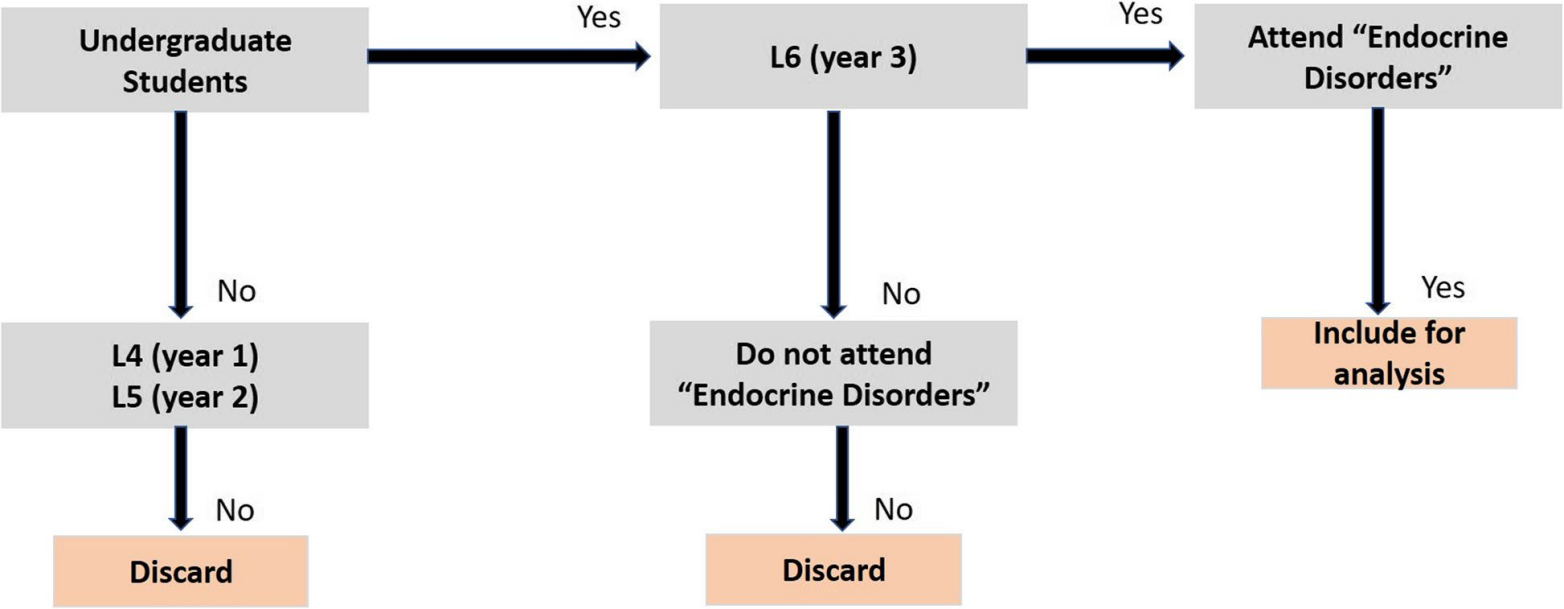
Flow chart of the experimental design and inclusion and exclusion criteria for the study. Of those included in the study, attendance/non-attendance in sessions were monitored, and the coursework and exam grades were determined for each group

**Fig. 2:**
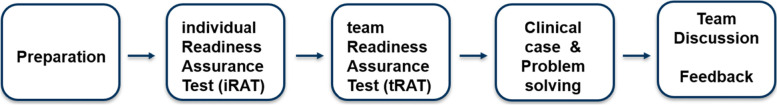
An overview of the administration of TBL and sequence of activities

Final year undergraduate students registered in the “Endocrine Disorders” study block were informed about the purpose of introducing non-mandatory TBL sessions in the first introductory lecture of this block. Participation in TBL sessions was not compulsory, and assessment of performance was formative. Figure 1 presents a flow chart of the experimental design and inclusion and exclusion criteria; Fig. 2 presents an overview of the applied administration of TBL.

### Preparation before class

As pre-session preparation, students opting to attend TBL sessions had to first attend a traditional 2-h lecture on a specific endocrine disorder prior to the corresponding TBL session. These lectures and the related teaching material (e.g. videos, research papers) were available via the university’s virtual learning environment (VLE) that is powered by the online platforms BlackBoard Learn and Panopto. Similarly, for the cohort that attended the class virtually, asynchronous material was uploaded before the lectures (synchronous sessions). Identical content was covered in both academic years presented in this analysis.

### In-person TBL

#### In-class readiness assurance testing (RAT)

Students - using the online tool “Poll Everywhere” on their mobile devices - had to complete an individual readiness assurance test (iRAT), which consisted of 10 multiple choice questions (MCQs) and lasted 5 min. After submitting the answers individually, students were asked to take the same test as a team (tRAT) for 10 min. As a team, students used scratch cards, hoping to find a star that indicates a correct answer. In this context, all members of each team shared the same tRAT score, whilst individual iRAT scores contributed as “points” to the overall performance of the team.

#### Application exercise

The remainder of each TBL session consisted of a problem-solving exercise/scenario, aiming to enable students to develop their cognitive skills through application of knowledge to the interpretation of clinical problems and case studies. Teams were allowed up to 20 min to discuss the problem or clinical case, after which they were presented with specific questions which they had to discuss and arrive at the best answer as a team. Teams were asked to display their selected answer, and the academic(s) facilitated discussion between teams to explore the reasoning and possible answers to the problem. This cycle was repeated until the problem had been solved; the purpose of building the overall solution through a series of questions is to make sure that students are guided in the right direction and do not lose motivation by being faced with a complex and difficult problem that they perhaps do not know how to tackle. Finally, scores were added to that of iRAT and tRAT to produce an overall mark for each TBL session.

### Online TBL

The use of a dedicated software enabled the implementation of the readiness assurance process and the application exercises synchronously online. Students joined the online session using the web conferencing platform Zoom, where they were able to interact with the lecturer and their peers. The students completed the iRAT using the software, and then were assigned to break-out rooms in Zoom where they proceeded to complete the tRAT in their TBL teams. The software provided real time data on the performance, facilitating the discussion back in the main Zoom room, once the readiness assurance process was completed. For the application exercises, students were put into their break out rooms again, where they discussed the problem or clinical case and input the team answers into the software application. When the allotted time was up, students were called back into the main room, where their answers were revealed simultaneously using the share screen feature in Zoom. The lecturer then had the opportunity to discuss the answers, inviting students to join in the discussion and explain their choices.

### Statistical analysis

Exam and coursework marks were analysed using student’s T-test with significance set at *p* < 0.05. Data are presented as means ± standard deviation (SD). Data analyses were performed using GraphPad Prism.

## Results

### Exam performance

**Fig. 3:**
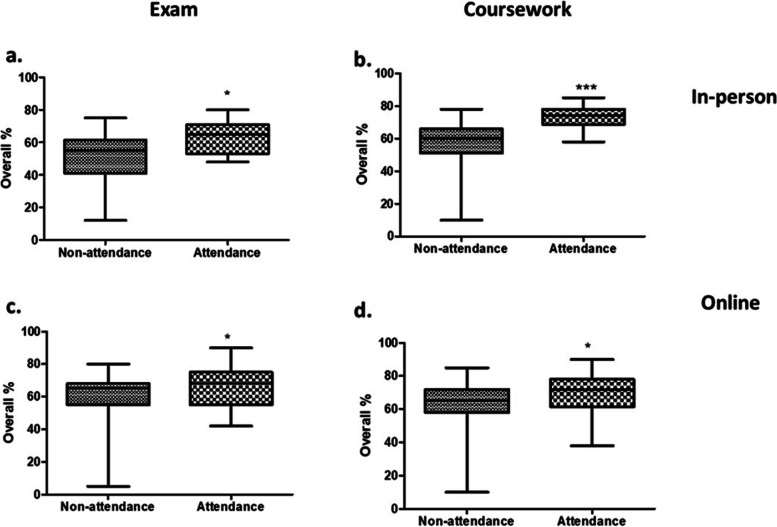
Compared to students who opted not to attend the TBL sessions (non-attendance), students who attended TBL sessions (attendance) performed better both in their final exams (**a**, **c**) and coursework (**b**, **d**) in two different cohorts attending in-person or online. *P < 0.05, *** *P* < 0.001

In the in-person cohort; 21 out of 66 students attended the optional TBL sessions (i.e. 31.8%). The students who attended TBL sessions had an overall performance of 63% and no fails for the final year examination, whereas students who opted not to attend the TBL sessions scored 50.4% (Fig. 3a). Moreover, all fails (i.e. E and F grades), as well as “no shows” were from the non-attendance group. Regarding coursework (a 1000-word written report for the module on “Problem Solving and Data Analysis”), the students who attended TBL sessions had an overall performance of 73.2% with no fails, whereas students who did not attend the optional TBL sessions scored 56.3% (Fig. 3b). In this coursework, students were given raw data from a clinical case relating to an endocrine disorder, and asked to produce an essay analysing the provided data. In doing so, they will have to demonstrate in-depth knowledge of subject learnt from the relevant study block, as well as independent thinking, analytical and problem-solving skills.

In the online cohort, 32 out of 109 students attended the TBL (i.e. 29.4%). The students who attended the online TBL scored on average 66.9% in their exams (and no fails), compared to 60.1% in the non-attendance group (Fig. 3c). Similarly, the cohort of students that attended TBL had an average of 69.1% in their coursework compared to 62.4% in the non-attendance group (Fig. 3d).

### Student satisfaction

**Fig. 4:**
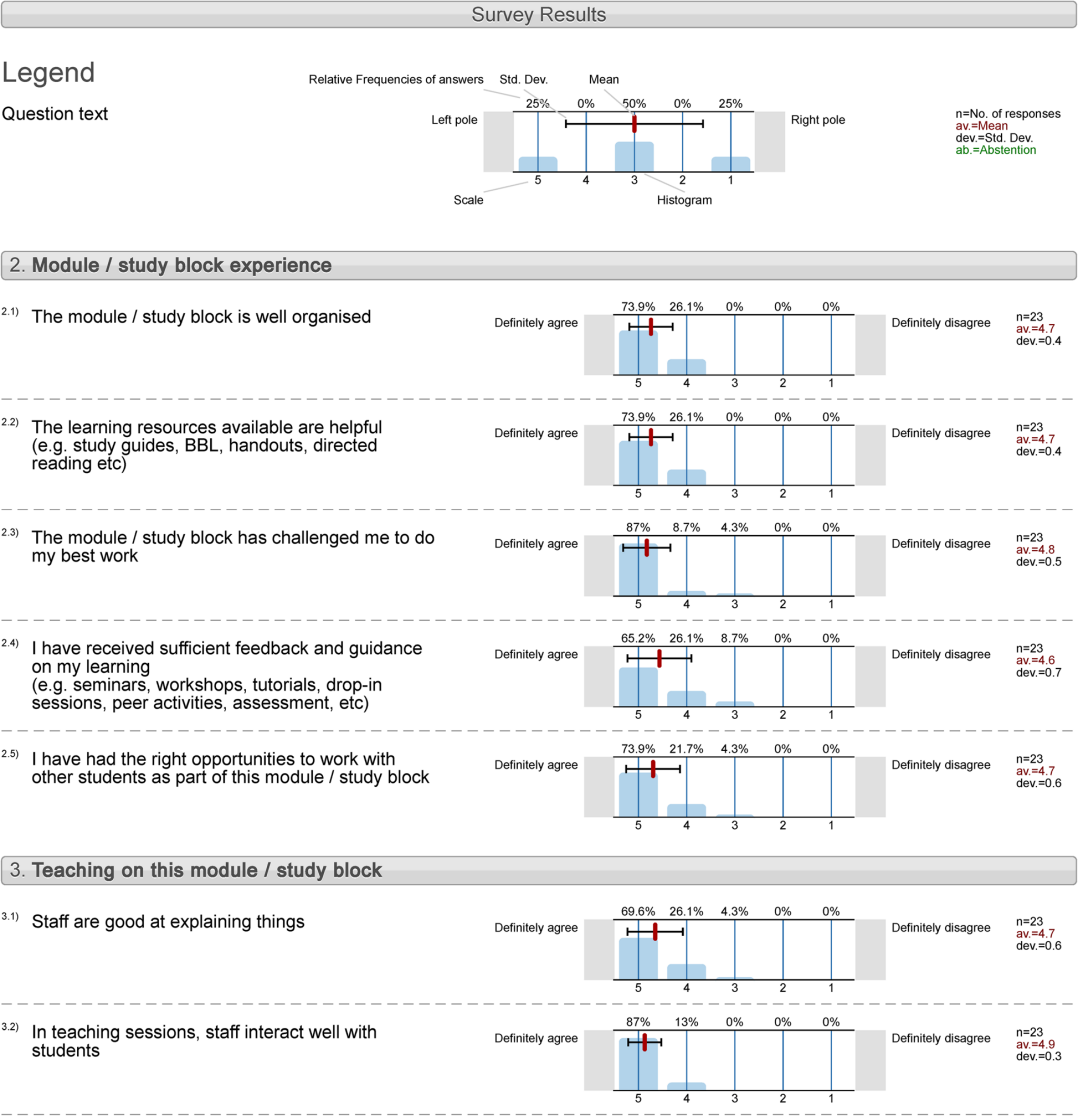
Feedback from students of in-person cohort on the “*Endocrine Disorders*” study block experience and teaching of this study block, using University’s online module survey, “*YourVoice*”

The applied TBL sessions also played a key role in “*YourVoice*” module evaluations from students. This is the university’s online module survey which offers all students the opportunity to provide their anonymized feedback on various aspects of their teaching and learning experience, as well as suggestions on potential improvements for each module/block, using a 5-points Likert scale. The “Endocrine Disorders” block in the present case-study scored 4.8/5.0 in the global teaching index, with the highest scoring (4.9/5.0) in the category for Study block experience under “*In teaching sessions, staff interact well with students*” (Fig. 4). In the cohort of students attending in-person TBL, 23 out 66 (34.8%) responded to the non-compulsory survey. The response rate of the 2020–2021 cohort in the “*YourVoice*” module evaluations was very low, with only 9 out of 109 students (8.3%) completing the questionnaire, so no safe conclusions can be drawn, although the response was similar (data not shown).

This survey, also contains a “*Comments Report*” section, where students can provide free-text, qualitative feedback on the study block in the following question: “*What were the most positive, helpful or enjoyable aspects of this module/study block?*”. cohort replies to this included:

- “*Definitely the TBL sessions! Honestly would recommend having more of them. I know for sure my group agree, everybody wanted to do more because we all saw how much it contributed to our learning. Teachers were amazing. Felt for the first time that I actually interacted with lectures in a positive manner... The timetabling was perfect; lecture then TBL. Gives you a chance to revise the topic before and then have a recap of the whole thing in an active learning scenario. Can't praise it enough.*”
- “*Overall, I have an extremely positive experience with this module, the TBL sessions mixed with the lectures made the subject easier to comprehend and so much more interesting. The case studies (during TBL) allowed us to work together with a problem and develop our critical thinking as well as discussing with each other about the topics we have been taught.*”
- “*The TBL sessions were amazing!! I think other modules should definitely do this!*”
- “*The TBL sessions were amazing and really helped me cement my knowledge and put it into use to evaluate the case studies. Also this is a good way of actually preparing for the exam, because it shows clearly how we need to use and apply our knowledge.*”

Similar comments were received from the online cohort, commending the online TBL sessions:

- “*I really enjoyed this module - the integrated look at the endocrine system from the top-level medical overview... Very integrated and highly enjoyable. The TBL sessions were also excellent and would’ve been better if they hadn’t been virtual, but what can you do, that’s COVID-19.*”
- “*TBL Sessions have been very helpful as well as review articles we have been given.*”
- “*The TBL sessions and lectures particularly focusing on case studies were most helpful.*”

## Discussion

Despite the adoption of TBL in HE worldwide, there is limited data on its application in HPE and particularly in the field of Endocrinology. Indeed, a recent systematic review of the relevant existing literature identified significant gaps regarding how TBL can be utilised on HPE settings other than medicine, including nursing, pharmacy, and occupational therapy [10]. The present study offers new evidence to address such gaps regarding incorporating TBL in the curriculum of biomedical sciences for complex and clinically-oriented blocks/modules. As such, our case study demonstrates that attendance of structured TBL sessions can enhance the overall academic performance of the students in both coursework and exams, irrespective of the mode of delivery (in-person or online). This is in line with findings of a US study on the adoption of TBL in a pharmacy curriculum, including endocrinology as a subject, which showed that this approach improved students’ clinical and teamwork skills, and gave them the opportunity to prepare better for examinations [25]. Similarly, when a TBL approach was adopted to create different modules on diabetes (one of the most common endocrine disorders), teams outperformed individuals on the tests by 18% [12]. It should be noted that in our cohort of students, there were no obvious differences in the previous year’s exam performance between those who attended TBL sessions in 2019/20 and those that did not (data not shown), which suggests that the better outcomes for students attending TBL sessions is an effect of the TBL learning experience, and not because they were an academically better self-selected sub-group.

The TBL sessions also played a key role in the anonymous module evaluations/feedback from students. The “Endocrine Disorders” block in the present case study scored particularly high in the global teaching index (4.8/5.0), with the highest scoring (4.9/5.0) in the category for “*In teaching sessions, staff interact well with students*”. These data are in direct agreement with recent studies on the impact of TBL on students’ satisfaction [26]. For example, the majority of students from a US college of Pharmacy agreed that creating TBL modules enhanced their understanding of concepts (76.7%), improved their self-directed learning skills (86.7%), as well as their comprehension of this pedagogical tool (90%) [27]. Overall, the available evidence suggests that students who attended TBL sessions were able to become confident and articulate in scientific reasoning, developed the ability to evaluate relevant literature, and understand the complexity and interrelationships of different scientific disciplines [26].

As mentioned previously, in response to the COVID-19 pandemic, the delivery of TBL sessions for the “*Endocrine Disorders*” study block was moved online. Although the online TBL attendance was marginally lower to that of the in-person cohort TBL (29.4% vs 31.8%, respectively), overall our data demonstrates that TBL can enhance the learning/performance, irrespective of the mode of delivery. Thus, our present findings support the feasibility and potential of online TBL, that can be deployed to facilitate remote learning for such complex subjects [27–31]. However, it should be noted that there were a number of challenges for the online delivery, including being proficient on the use of different online platforms, co-ordination with facilitators while delivering the session, reliability of internet networks, and inability to observe the class as a whole (moving to online break-out rooms instead). We should also acknowledge that the small scale of the present case study is a limitation for the generalization of our findings for the delivery of TBL in the context of complex Biomedical Sciences’ subjects/modules. Finally, further demographic details including age, sex or analysis on the performance of black, asian and minority ethnic (BAME) students, can provide a deeper insight into the benefits of the use of TBL.

## Conclusions

In conclusion, we show that TBL -online or in person- can increase the performance of final year undergraduate biomedical students for an Endocrinology-related module that delivers complex and clinically relevant subject content. Adoption of online TBL delivery can facilitate remote learning whether it is to facilitate learning under conditions such as those imposed by the current COVID-19 pandemic, or to accommodate students that study part-time. We propose that TBL should be incorporated in the curriculum of HPE as a useful pedagogical method to enhance the learning experience and improve the overall performance of undergraduate students reading for BSc degrees in the biomedical field.

## Data Availability

The datasets used and/or analysed during the current study are available from the corresponding author on reasonable request.
